# Effect of Organo-Modified Nanoclay on the Thermal and Bulk Structural Properties of Poly(3-hydroxybutyrate)-Epoxidized Natural Rubber Blends: Formation of Multi-Components Biobased Nanohybrids

**DOI:** 10.3390/ma7064508

**Published:** 2014-06-13

**Authors:** Ali Salehabadi, Mohamad Abu Bakar, Noor Hana Hanif Abu Bakar

**Affiliations:** Advanced Material Research Laboratory, School of Chemical Sciences, Universiti Sains Malaysia, Penang 11800, Malaysia; E-Mails: asn10_che012@student.usm.my (A.S.); hana_hanif@usm.my (N.H.H.A.B.)

**Keywords:** PHB, ENR-50, orgnomodified nanoclay, nanohybrid, thermal behavior

## Abstract

Multi-component nanohybrids comprising of organo-modified montmorillonite (MMT) and immiscible biopolymer blends of poly(3-hydroxybutyrate) (PHB) and epoxidized natural rubber (ENR-50) were prepared by solvent casting technique. The one and three dimensional morphology of PHB/ENR-50/MMT systems were studied using Polarizing Optical Microscopy (POM) and Scanning Electron Microscopy (SEM). Differential scanning calorimetry (DSC) technique was used to evaluate the thermal properties of the nanohybrids. The melting temperature (*T*_m_) and enthalpy of melting (Δ*H*_m_) of PHB decrease with respect to the increase in ENR-50 as well as MMT content. The non-isothermal decomposition of the nanohybrids was studied using thermogravimetric (TG-DTG) analysis. FTIR-ATR spectra supported ring opening of the epoxide group via reaction with carboxyl group of PHB and amines of organic modifier. The reaction mechanism towards the formation of the nanohybrids is proposed.

## 1. Introduction

The study of multi-component organic-inorganic hybrids bears prominent challenges in most scientific articles. Based on bulk structural study, the interfacial structure and dynamic properties are two important issues, which govern the type of interactions between the polymer matrix and the embedded fillers [1]. An overview of previous published articles shows that polymeric materials filled with nanoscale platelet layered silicates, especially montmorillonite (MMT), have received more attention due to their improved mechanical and thermal properties [2,3,4,5]. Stacking of montmorillonite layers occurs through weak van der Waals forces, and can be expanded by polymer intercalation into the silicate layers which can be further dispersed in the polymer matrix [6]. The basal spacing of MMT, comprising the platelets separated by an interlayer gallery, can vary from fully collapsed to more expanded state. To reduce the polar characteristic of the silicate, cation exchange reactions have been applied with various organic modifiers to render the hydrophilic silicate surface tendency to partial organophilic character [7].

Biodegradable polymer blends and nanocomposites [8,9,10,11,12,13,14,15,16], as novel environmental-friendly materials, have attracted great interest due to environmental concern. Since biopolymers are obtained from renewable resources, they possessed advantages over the non-degradable polymers over a range of applications like packaging, agriculture, medicine, *etc.* Poly (3-hydroxybutyrate) (PHB) is a type of biodegradable and biocompatible polymers suitable for relieving environmental concerns and providing new type biomedical products [3]. A number of deficiencies, like high crystallinity of bacterial PHB, instability in the molten state, and high production cost limit the applications of this polymer [17,18]. To overcome these drawbacks, PHB has been subjected to polymer-polymer blending (either miscible or immiscible), and materials loading (polymer-filler composites) and the likes. 

There are few studies regarding nanocomposites comprised of immiscible bio-based polymer blends. It has been demonstrated that most of the polymer blends are immiscible and tend to separate into macroscopic phases and reveal clear individual glass transition temperatures [3,8,9,19]. Lee *et al.* [20] reported that in blends of PHB/ENR-50, the carboxyl end group of degraded PHB and the epoxide group of ENR are reactive and may affect chemical and physical, as well as structural changes at the interfaces of the immiscible PHB and ENR phases. They also pointed out that the extent of miscibility is more pronounced in molten state. In the current study, with respect to our previous studies on PHB/MMT [21] and ENR-50/MMT nanohybrids [22], we prepare multi-component nanohybrids comprising of multi-component PHB/ENR-50 biopolymer blends containing various wt% amounts of MMT. Three compositions of PHB/ENR-50 (viz. 30/70, 50/50 and 70/30) were selected based on the morphology and extent of miscibility of the polymer domains in the blends as previously reported [19]. These compositions were employed to study the effect of MMT on properties of the PHB/ENR-50 blends which are described herewith.

## 2. Results and Discussion

### 2.1. Surface Analysis and Bulk Structure

**Figure 1:**
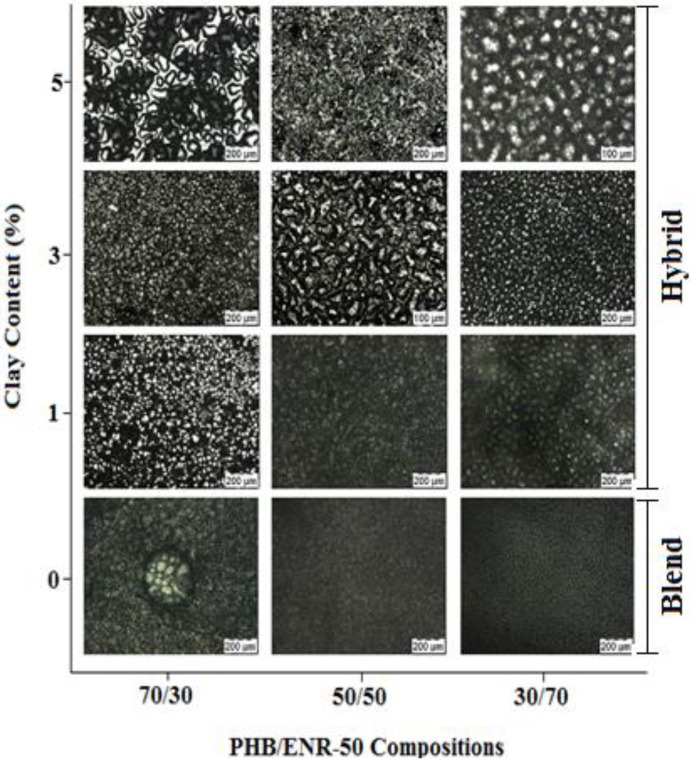
Polarized Optical Micrographs of various PHB/ENR-50 blends and the respective blends containing various wt% MMT at room temperature.

Figure 1 shows POM images of various compositions of PHB/ENR-50 blends and the various blends containing 1, 3 and 5 wt% MMT. The PHB/ENR-50 blends show different optical characteristics. When the 30/70 PHB/ENR-50 blend was tested, fraction of PHB is still observed. However, upon increasing the fraction of PHB in the polymer blend (*i.e.*, 50/50 blend), a regular but co-continuous morphology is obtained. Further increase in the fraction of PHB (as in 70/30 blend) causes a phase inversion whereby the PHB assumes the matrix and ENR-50 is the dispersed phase. Thus, by increasing the PHB fraction in the blends, clusters of PHB crystals occur especially in the 70/30 composition of PHB/ENR-50. In contrast, upon increasing the fraction of ENR progressively, the morphology of the blend shows a homogeneous structure due to the surface covering of the PHB crystals by ENR-50 domains.

When MMT is incorporated into the blend with a lower fraction of PHB, uneven distribution of nanoclay in the polymer matrix is observed. This is attributed to the agglomerations of clay particles in the blend. This phenomenon is more apparent in PHB/ENR-50 blends containing 5 wt% MMT. However, when equal fraction of amorphous and crystalline domain is employed, the 1 wt% MMT is dispersed homogeneously with narrow distribution of nanoclay within the polymer matrix. It seems at this blend composition and low MMT content, the extent of interaction of blend-MMT is less. As a result, the MMT particles were distributed freely within the blend. A packed and uneven morphology are instead observed for blends containing 3 and 5 wt% MMT respectively. On the other hand, by increasing the PHB fraction, the blend becomes more brittle, the MMT agglomerates into islands and the continuous surface are cracked. An obvious appearance of crack can be observed in the 70/30 composition of PHB/ENR-50 containing 5 wt% MMT.

**Figure 2:**
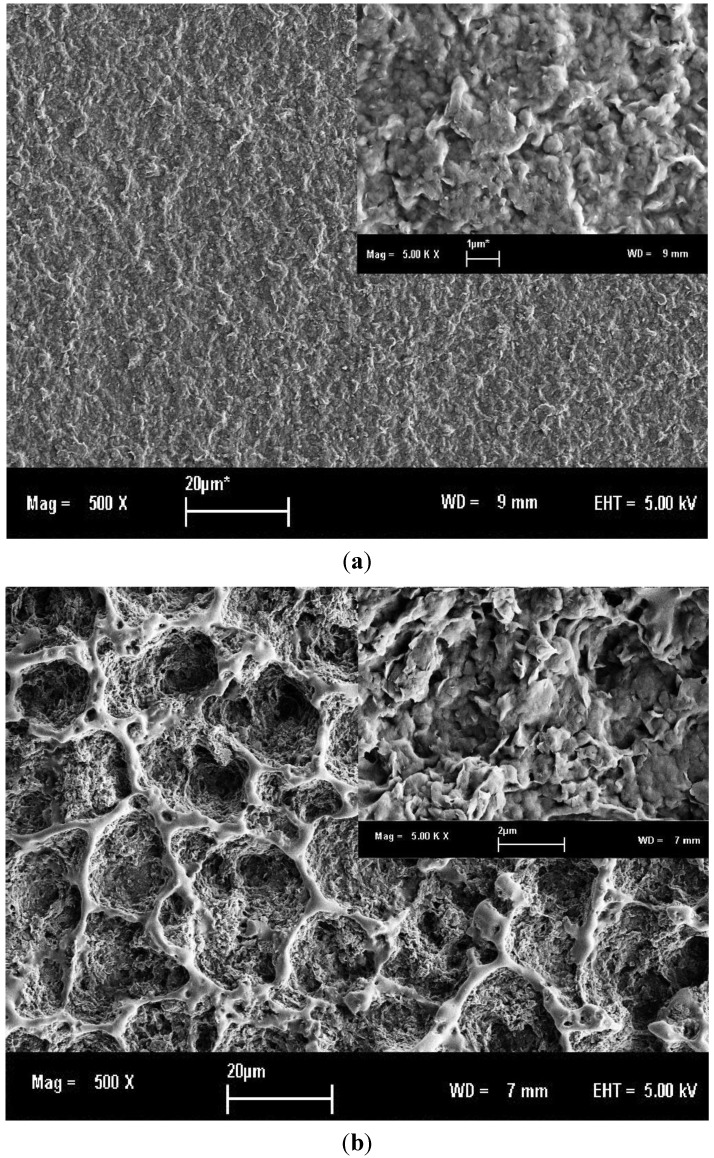
SEM micrographs of equal fraction of PHB/ENR-50 blend containing (**a**) 3 and (**b**) 5 wt% MMT (both at 5 kV with 500× and inset: 5000× magnifications).

Figure 2 compares the surface morphologies of 50/50 composition of PHB/ENR-50 blends containing 3 and 5 wt% MMT. In this blend, it is observed that upon addition of 3 wt% MMT, packed and folded morphology is formed. The interfaces are dispersed in the internal galleries of silicate layers. In blend containing 5 wt% MMT, some obvious interface can be observed.

**Figure 3:**
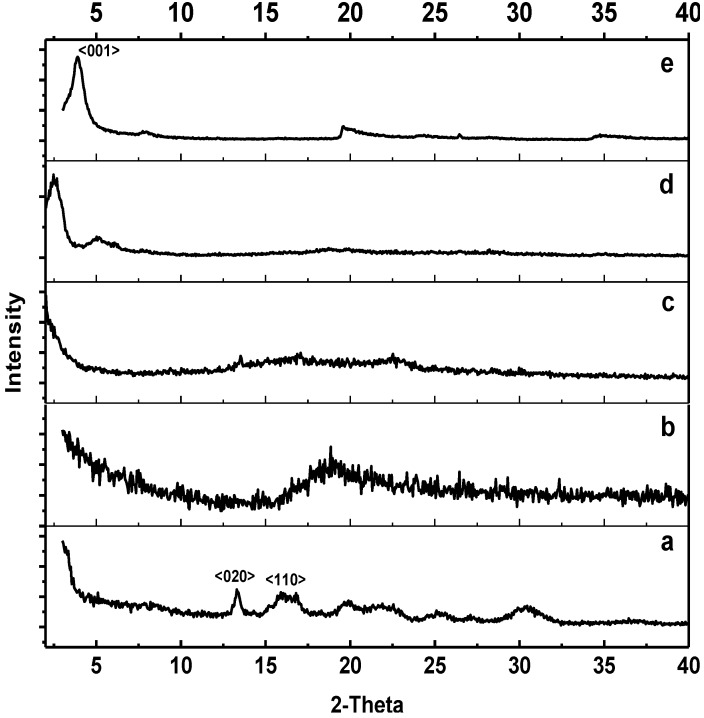
XRD patterns of (**a**) PHB; (**b**) ENR-50; (**c**) PHB/ENR-50 (50/50) blends; (**d**) PHB/ENR-50 (50/50) blends containing 5 wt% MMT; and (**e**) MMT.

Figure 3 shows the X-ray diffraction patterns of PHB, ENR-50, PHB/ENR-50 (50/50) blends containing zero and 5 wt% MMT and pristine MMT. The diffractogram of PHB is shown in Figure 3a. This polymer exhibits two intense peaks at 2θ values of 13.33° and 16.05°. These peaks are assigned to the <020> and <110> planes of the orthorhombic unit cell of PHB and is in agreement with those reported by Holmes [23] and Zihijiang *et al.* [24]. In contrast, ENR-50 does not show any peaks due to the amorphous nature of the polymer as observed in Figure 3b. The diffractogram of pristine MMT shown in Figure 3e demonstrates several peaks. These peaks are positioned at 2θ values of 3.913°, 7.869° and 19.511° corresponding to an interlayer distance of 2.256 nm, 1.123 nm and 0.455 nm respectively.

Introducing an amorphous domain of ENR-50 into the crystalline PHB has resulted in disordering of the crystal arrangements of the later polymer. This is depicted by the fact that majority of the PHB peaks disappeared as shown in Figure 3c. This also suggests a well dispersion of ENR-50 and dominance in PHB. Incorporation of 5 wt% MMT into 50/50 PHB/ENR-50 blend (Figure 3d) resulted in the MMT peaks to appear at 2θ values of 2.535° and 5.060°. This corresponds to the interlayer spacing of 3.484 nm and 1.746 nm respectively. There is a slight shift in peak position when compared to pristine MMT that suggests a proper intercalation of polymer chains into the silicate layers. According to Vu *et al.* [25] and Botana *et al.* [3], the decrease in peak intensity was due to the swelling of the alkyl ammonium modified nanoclay in organic solvents. Thus, in this work, introducing a polymer solution into the clay layers was sufficient to cause intercalation of the polymer into the galleries of the swelled clay and expand the MMT intergalleries. This is depicted by the shift of the peaks to lower d-spacing values when compared to pristine MMT [21].

### 2.2. FTIR Spectroscopy and Proposed Mechanism

**Figure 4:**
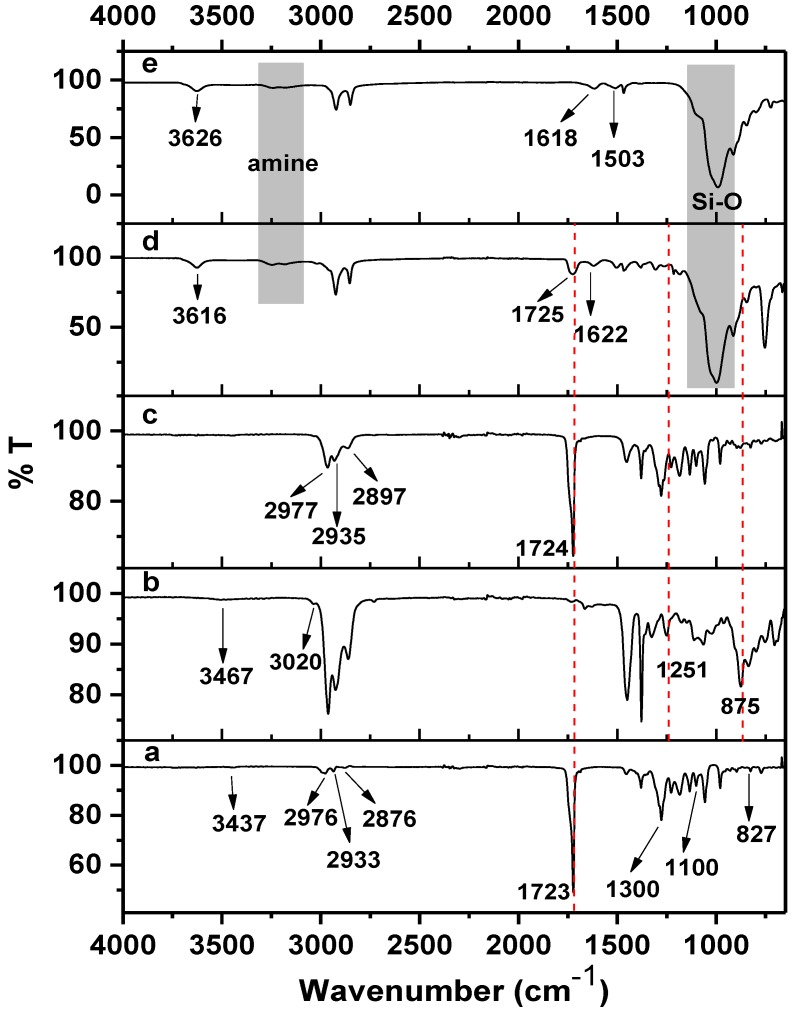
FTIR spectra of (**a**) PHB, (**b**) ENR-50, (**c**) PHB/ENR-50 (50/50) blend, (**d**) ENR/PHB (50/50) blend containing 5 wt% MMT and (**e**) MMT.

**Scheme 1:**
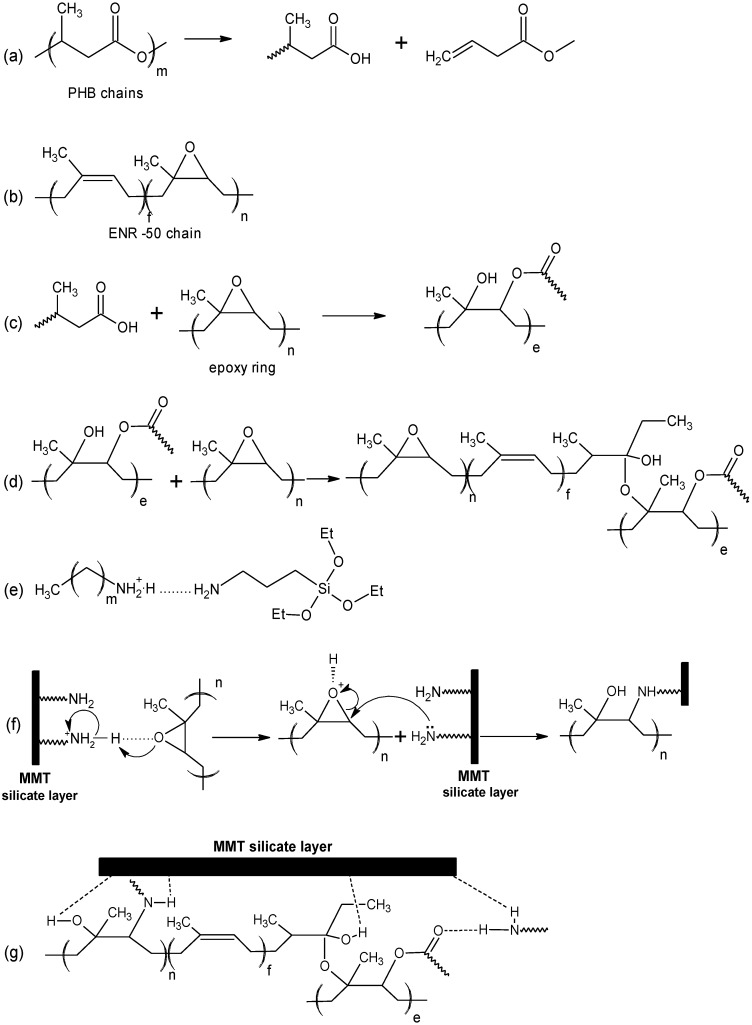
Proposed mechanism for the formation of PHB/ENR-50/MMT nanohybrid.

The FTIR spectra of PHB, ENR-50, PHB/ENR-50 (50/50) blend and the respective nanohybrid comprising of the (50/50) blend containing 5 wt% MMT and MMT are shown in Figure 4. Some of the FTIR characteristic bands are tabulated in Table 1. On the basis of current FTIR spectra, the proposed reactions and mechanism of formation of the nanohybrids are shown in Scheme 1.

In the spectrum of PHB (Figure 4a), a very strong band at 1723 cm^−1^ is due to carbonyl and the –C–O stretching of ester is at 1101 cm^−1^. The characteristic –OH stretching band can be observed at 3437 cm^1^. This arises due to the chain scission of PHB to shorter chain lengths [3,15] creating more carboxyl functionalities as shown in Scheme 1a.

The spectrum of ENR-50 (Figure 4b) shows the stretching vibrations of epoxy and half epoxy at 1251 and 875 cm^−1^ respectively [26]. Moreover, a strong absorption band can be observed at 3020 cm^−1^ which is due to the –C=CH of isoprene. The structure of ENR is shown in Scheme 1b.

The spectrum of the equal fraction of PHB/ENR-50 blend (Figure 4c) shows the –C–O stretching of ester appears at 1100 and 1305 cm^−1^. A strong band at 1724 cm^−1^ and a weak broad band at 877 cm^−1^ are respectively assigned to the carbonyl and epoxy that obviously indicates a different environment in the final blend. The characteristic band of epoxy becomes broad and weak most probably due to the lowering in epoxy content in the final blend caused by epoxy ring opening reactions. It is proposed that the hydroxyl (of carboxyl) of PHB reacts with the epoxy ring by attacking the less hindered carbon of the latter (Scheme 1c). This can further react with the remaining epoxy to ring open or terminate the reaction (Scheme 1d). Therefore a new blend structure (or new polymer product) can be assumed which still contains carbonyl and but a lesser number of epoxy ring. The same reaction has been proposed by Lee *et al.* [20] on the basis of melt reaction between two respective immiscible polymers.

**Table 1:** FTIR adsorption bands of PHB, ENR-50, PHB/ENR-50 (50/50) blends, the respective nanohybrid containing 5 wt% MMT and MMT.

Functional Group	PHB	ENR–50	Blend	Nanohybrid	MMT
(CO)–OH	–	–	3684,3621	–	–
Si–OH	–	–	–	3616	3626
–OH	3437	–	3464	3436	3428
Free Amine	–	–	–	3243,3182	3265,3175
–C=CH	–	3020	3019	–	–
Methyl	2976,1378,827	2973,1380	2977	2977–2895 *	–
Methylene	2933,1453	2912	2935	2928–1466	2921,1468
Methine	2876	2853	2897	2856	2851
–(O)C=O	1723	–	1724	1725	–
–C–O–	1300,1100	–	1305,1100	–	–
Epoxy	–	1251,875	1218,877	–	–
–Si–O	–	–	–	1100–915	1100–915
–C=C–	–	759,669	1602,760,672	1667	–
–NH_2_^+^	–	–	–	1622	1618
–NH_2_^+^Amine	–	–	–	1503	1503

* Overlapping peaks.

The spectrum of the PHB/ENR-50 (50/50) blend containing 5 wt% of organomodified MMT (Figure 4d) shows interesting features. Understanding of such a reaction needs apparent knowledge of clay structure after modification. From the spectrum, carbonyl shows a strong band at 1725 cm^−1^. The displacement and broadening of carbonyl band as compared to the pristine blend (Figure 4c) and PHB (Figure 4a) is most probably due to the H-bonding and the restriction effect of the silicate layers, respectively.

It is logical to assume that the silicate layers of MMT hinder the various polymer chain motions. This is supported by the fact that, nearly all the band intensities decrease as MMT loading is increased [21]. Furthermore, the disappearance of two characteristic bands of epoxy at 1251 and 875 cm^−1^ confirms the epoxy ring opening reaction. The ring opening reaction occurs due to the free amine groups present in the clay structure. The formation of covalent bond via ring opening reaction is shown in Scheme 1f. In this structure, a new bond –N–C– bond is formed. Scheme 1g indicates the proposed structure of the new nanohybrid comprising of the reactive polymer blend and organomodified MMT. The structure is also affected through various H–bondings.

In this study, the MMT nanoclay is modified with two organic modifiers containing primary amine groups. Many opinions have been expressed by researchers to explain the reaction of the amine groups with each other and with silicate layers as well [7]. In the interlayer space within the MMT, the ammonium ions can be generated in two ways; either by cation exchange or by protonation. In the presence of an amine compound, an ammonium-amine association can be obtained [7]. The spectrum of MMT (Figure 4e) confirms the ammonium-amine complex formation with the apprearence of a band at 1503 cm^−1^. There are two adsorption bands in the 3265 and 3175 cm^−1^ region attributed to the –NH stretching vibration. Scheme 1e depicts the formation of ammonium-amine complex between octadecyl ammonium cation and aminopropyltriethoxysilane [7]. The extent of the reaction is highly dependent to the amount of the organic components present and from the spectrum there are still some free amines remaining after the reaction.

### 2.3. Thermal Behavior

**Figure 5:**
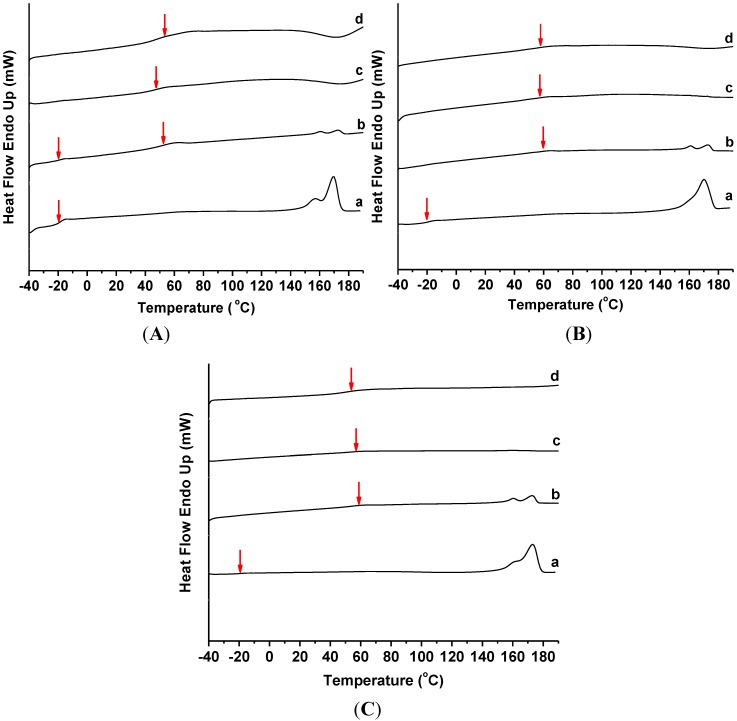
DSC thermograms (first heating program) of PHB/ENR-50 blend composition of (**A**) 30/70; (**B**) 50/50; and (**C**) 70/30 containing (a) 0, (b) 1, (c) 3, and (d) 5 wt% MMT.

Figure 5 exhibits the DSC profiles of the various PHB/ENR-50 blends containing 0, 1, 3 and 5 wt% MMT based on the first thermal/heating program and the results are summarized in Table 2. The double melting endotherms of PHB/ENR-50 blends as observed in Figure 5A–C(a) are due to polymorphism of PHB. Increasing the fraction of ENR-50 in the blends caused the melting temperature (*T*_m_) and enthalpy of melting (∆*H*_m_) to decrease. As shown in Table 2, addition of 1 wt% MMT to the blend also caused the *T*_m_ to decrease. However, further addition of MMT (*i.e.*, 3 and 5 wt%), caused these two corresponding temperature peaks to disappear. This is presumably due to the alteration in the PHB crystal structure by the increase presence of MMT.

The glass transition temperature (*T*_g_) of ENR-50 can be observed in the various PHB/ENR-50 blend compositions. These are assigned as *T*_g1_ in Table 2. The second *T*_g_ (*T*_g2_) is due to the glass transition temperature of organomodified montmorillonite.

**Table 2:** Thermal transition (DSC) and thermal decomposition (TG-DTG) data of PHB/ENR-50 (30/70, 50/50 and 70/30) blends and respective nanohybrids containing 1, 3 and 5 wt% MMT.

PHB/ENR-50 composition	Clay content (wt%)	DSC *				TG-DTG **				
		*T*_g1_ (°C)	*T*_g2_ (°C)	*T_m_* (°C)	∆*H*_m_ (J/g)	*T*_10_ (°C)	*T*_20_ (°C)	*T*_30_ (°C)	*T*_max1_ (°C)	*T*_max2_ (°C)
30/70	0	−18.1	–	169.6	25.8	255.6	281.5	370.4	280.9	433.1
	1	−18.8	53.5	172.4	4.1	270.4	318.5	366.7	332.8	438.2
	3	–	49.3	–	–	296.3	366.7	403.7	310.9	432.7
	5	–	60.0	–	–	314.8	377.8	414.8	291.0	430.7
50/50	0	−17.7	–	170.2	37.5	248.2	259.3	270.4	279.1	437.8
	1	–	60.7	172.4	9.1	266.7	281.5	307.4	313.9	431.5
	3	–	57.3	–	–	274.1	340.7	388.9	300.0	429.5
	5	–	58.6	–	–	285.2	388.9	425.9	294.4	441.0
70/30	0	−19.3	–	173.1	53.7	235.2	242.6	248.2	276.9	414.2
	1	–	58.0	172.8	18.3	281.5	292.6	300.0	303.6	446.7
	3	–	57.4	170.0	1.9	274.1	296.3	377.8	297.9	438.7
	5	–	54.3	–	–	266.7	355.6	407.4	284.1	429.5

**Figure 6:**
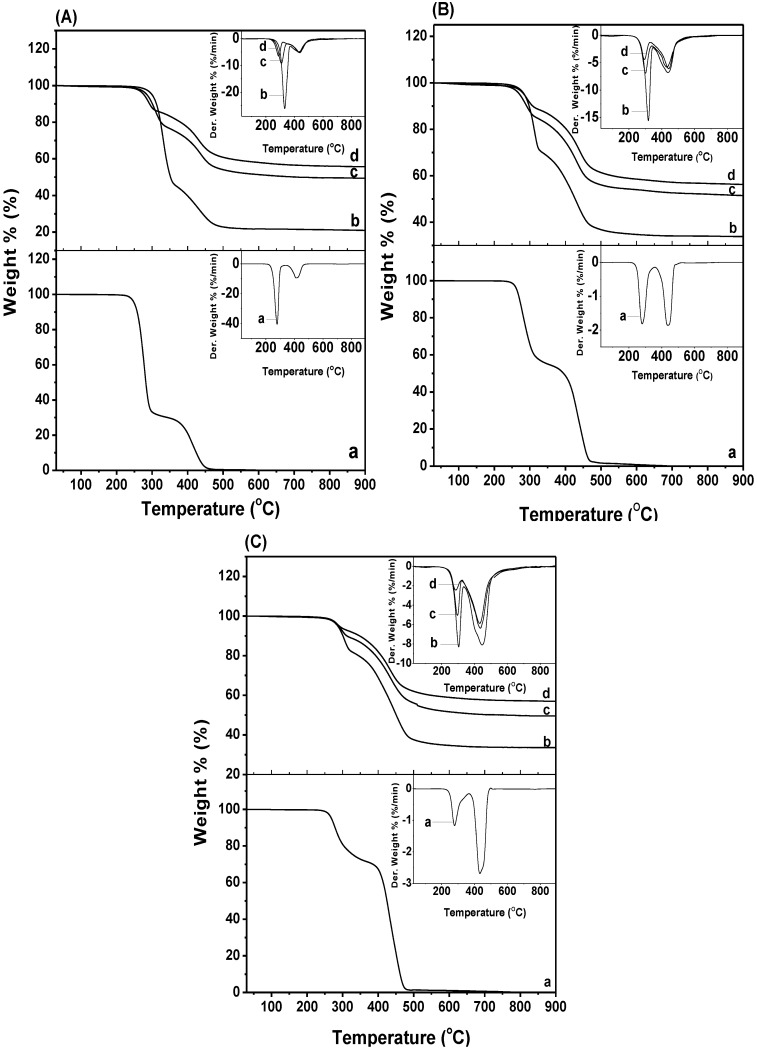
TG-DTG curves of PHB/ENR-50 blend composition of (**A**) 70/30; (**B**) 50/50; and (**C**) 30/70 containing (**a**) 0, (**b**) 1, (**c**) 3, and (**d**) 5 wt% MMT.

* and ** refers to the plots of Figure 5 and Figure 6, respectively. *T*_g1_ and *T*_g2_ represent the 1st and 2nd glass transition temperature from 1st heating program observe in DSC; *T*_10_, *T*_20_ and *T*_30_ represent the degradation temperatures at 10%, 20% and 30% mass loss, respectively; *T*_max1_ and *T*_max2_ represent the maximum mass loss temperature observe in DTG thermograms.

Figure 5A shows the DSC thermograms of 30/70 PHB/ENR-50 blend containing 1, 3 and 5 wt% MMT. Increasing the MMT content increased the second *T*_g_ (*T*_g2_). However, the trend is not linear. This is because the affinity of interactions between polymers and organoclay. However, in PHB/ENR-50 blends comprising equal or reduce fraction of ENR-50 (*i.e.*, 50/50 and 70/30) with similar MMT organoclay content (Figure 5B,C), the *T*_g2_ was displaced to a lower temperature. This is due to the reduced ENR-50/MMT organoclay interactions coupled with lower affinity of PHB towards MMT.

It seems that in the blends with higher composition of PHB, the organomodified nanoclay imparted catalytic effect [21] and barrier effect is dominant in the blends with higher ENR-50 [22]. Therefore, the *T*_g2_ decreases and allows the polymer to achieve the rubbery state at a lower temperature. 

In this work, all of the PHB/ENR-50/MMT nanohybrid thermograms showed a single *T*_g_ except the PHB/ENR-50 (30/70) containing 1 wt% MMT. In fact, the *T*_g_ is a function of miscibility of the polymers and the extent of reaction of the blend components can therefore be deduced on the basis of *T*_g_. Previous study on PHB/ENR-50 blends [19] have shown that various compositions of blend compartments exhibit different extent of miscibility.

### 2.4. Thermal Decomposition

Thermal decomposition profiles of the various PHB/ENR-50 blends and the respective blend/MMT nanohybrids are shown in Figure 6. Their respective decomposition temperatures are tabulated in Table 2. In the pristine blends (Figure 6A–C(a)), upon increasing the ENR-50, the first (*T*_max1_) and second (*T*_max2_) maximum were slightly displaced. It is obvious that the *T*_max2_ of 50/50 PHB/ENR-50 blend are shifted to the higher temperature as compared to the blends with a composition of 70/30 and 30/70. The higher displacement of *T*_max_ can be due to the better dispersion of the PHB and ENR-50 in this composition. It must be mentioned that the thermal decomposition of pristine PHB and ENR-50 occurs in single stage at 288.8 °C and 418.8 °C, respectively [21,22].

Increasing the MMT content in various PHB/ENR-50 blends caused the degradation temperatures at 10%, 20% and 30% mass loss to shift to higher temperatures. The degradation temperatures of the nanohybrids are higher than the polymer blends itself and can be attributed to the barrier effect of MMT which inhibits heat transfer to the polymer blends. This is true for all the series studied except for the hybrid comprising of low fraction of ENR-50 (*i.e.*, 70/30 PHB/ENR-50; Figure 6A). Here at 10% mass loss, a decrease in the degradation temperature as the MMT content is increased from 1–5 wt% is observed. This variation in trend can only be explained as due to the fact that MMT promotes catalytic decomposition of PHB in the samples [27].

Another observation is that two *T*_max_ occur in all the hybrid samples and are tabulated in Table 2. The *T*_max1_ is due to the PHB while the *T*_max2_ is attributed to the decomposition of ENR-50. The *T*_max_ values decrease upon increase in MMT content from 1 to 5 wt% for all the series investigated except the *T*_max2_ of the hybrid comprising 50/50 PHB/ENR-50 with 5 wt% MMT. The exceptional *T*_max2_ value for the hybrid comprising of 50/50 PHB/ENR-50 and 5 wt% MMT content can be explained as due to the agglomeration of MMT particles. 

Nonetheless, all the *T*_max_ values are superior as compared to the pristine PHB/ENR-50 blends. Generally, the decrease in *T*_max_ value upon increase in the MMT content in the hybrid is due to the catalytic effect of Lewis acid sites in the MMT [28]. In the thermal treatment, the organomodifiers viz. alkyl ammonium salts, the ammonium cation degraded to olefin and amine. As a result, the available proton(s) impart catalytic effect during the initial stages of PHB decomposition and this is more apparent at high MMT loading [29]. It is therefore obvious that the decomposition temperature of the nanohybrids is much higher than respective pristine polymer blends. This improvement of thermal properties can be attributed to the production of a new compound that arises from proper reaction between the components.

Degree of delamination and dispersion can affect the thermal enhancement. Optimized processing conditions and proper polymer-clay interaction are two important factors affecting delamination and dispersion. Hence, the extent of interaction/reaction between polymer and clay layers either through the hydrogen bond or covalent bond formation contributes to this enhancement. These are shown in Scheme 1. It was proposed that the amine group presence on the surface of montmorillonite can form covalent bond. This occurs over the formation of –N–C– bond over the ring opening of epoxy groups thus creating the nanohybrid.

It is noted that upon increasing the MMT content in the samples, the ash content increase gradually. Here, the majority of organic compound decompose up to ~600 °C. At 900 °C, the char yields of the sample containing higher amount of MMT achieve higher value than that of the blend. The residue is mainly from inorganic MMT and components like Al_2_O_3_, MgO, and SiO_2_.

## 3. Experimental

### 3.1. Materials

Poly(3-hydroxybutyrate), PHB was from BIOCYCLE (São Paulo, Brazil) and ENR (50 mol% epoxidized, denoted as ENR-50) was from Guthrie Polymer Sdn. Bhd. (Siliau, Malaysia). Both were purified before use. The purification steps for PHB and ENR-50 involved dissolution of the polymer in chloroform, followed by filtration, and finally precipitation in a non-solvent [21]. Nanoclay (Nanomer 1.31PS, montmorillonite clay surface modified with 15%–35% octadecylamine and 0.5%–5% aminopropyltriethoxysilane; a product of Nanocor, Inc., AMCOL, Arlington Heights, IL, USA) was purchased from Aldrich Chemicals (SIGMA-ALDRICH, Co., St. Louis., MO, USA) and used as recieved. 

### 3.2. Preparation of Multi-Component Nanohybrid

Multi component nanohybrids based on PHB/ENR-50 blends of various compositions (70/30, 50/50, 30/70), and various wt% of organomodified montmorillonite (MMT) filler were prepared by solvent-casting from 1% (w/w) solution of PHB and ENR-50 in chloroform. This was followed by evaporation at room temperature for 24 h and finally drying in a vacuum oven at 40 °C for 24 h. Solvent-casting technique was chosen in this study to prepare homogeneous membranes. Three sets of experiments based on as mention blends with various MMT contents (viz. 1, 3 and 5 wt%) were performed.

### 3.3. Characterization

A Nikon Ellipse E600 (Nikon Eng. Co. Ltd., Yokohama, Japan) polarizing optical microscope (POM) equipped with a Linkam hot stage (Linkam Ltd., Surrey, UK) was used to obtain the POM micrographs of PHB/ENR-50/MMT nanohybrids. The morphology of the nanohybrids was determined using LEO Supra 50VP (Carl-Ziess, Oberkochen, Germany) Field Emission Scanning Electron Microscopy (FE-SEM) operating at an acceleration voltage of 5 kV. Typically, a sample was sputtered with gold using SC515 FISONS Sputter Coater (Fision Ins., VG microtech, Sussex, UK) prior to SEM investigations. 

Spectroscopic analysis of ENR-50, PHB, the various PHB/ENR-50 blends and PHB/ENR-50/MMT nanohybrids as well as MMT was carried out using FTIR Perkin-Elmer Frontier spectrometer. The sample was scanned on ATR mode in the range of 4000–650 cm^−1^. 

The X-ray diffraction (XRD) patterns of the samples were obtained in the 2θ range of 2° to 40° using the X’Pert Pro MRD PANalytical (PANalytical B.V., Almelo, Netherland) X-ray diffractometer. The basal spacing (d-spacing) was calculated using the Bragg Equation (1).

λ = 2*d*sinθ (1)

where λ is the X-ray wavelength; *d* is the basal spacing; and θ is the scattering angle.

A Perkin Elmer Pyris 1 DSC (PerkinElmer Inc., CA, USA) equipped with Perkin Elmer intercooler 2P as cooling accessory was used to investigate the glass transition (*T*_g_) and melting (*T*_m_) temperatures of the samples. Enthalpy of melting (Δ*H*_m_) was determined from the area of the endothermic peaks per gram of starting materials containing PHB (J/g). The first heating program was performed from −40 °C to 190 °C at 20 °C∙min^−1^ followed by quenching to −40 °C at a rate of 100 °C∙min^−1^. The sample was held at −40 °C for 5 min to reach an equilibrium state. The sample was then subjected to second heating where the samples were reheated to 190 °C at a heating rate of 20 °C∙min^−1^. Thermogravimetric (TG) analysis was conducted using Perkin-Elmer TGA 7 (PerkinElmer Inc., Newark, CO, USA). The samples were heated from 30 °C to 900 °C at a heating rate of 20 °C∙min^−1^ under nitrogen flow. 

## 4. Conclusions

Bio-nanohybrids comprising of nanoscale platelets derived from layered silicates (MMT) modified with alkyl ammonium organic modifiers and PHB/ENR-50 blends were prepared successfully by solvent casting technique. The increase in the interlayer spacing and expansion of silicate layers within the MMT in the blend were indicative of the formation of the nanohybrids. The basal spacing (d_001_) increases from 2.26 nm in pristine MMT to 3.48 nm in the case of the nanohybrids. The morphology based on the overall view of the bulk structure of the samples shows that the best structure occurs in PHB/ENR-50/MMT nanohybrids containing equal fraction of polymers and incorporated with 3 to 5 wt% MMT. Melting temperature (*T*_m_) of the nanohybrids is highly affected by blend composition. By increasing the ENR-50 fraction in blend and MMT content in the nanohybrids, the *T*_m_ achieves higher value. The glass transition temperature (*T*_g_) however, decreases upon increasing the content of PHB and MMT. The enthalpy of melting (∆*H*_m_) of PHB/ENR-50/MMT nanohybrids decreases as ENR-50 and MMT content is increased due to lowering in the crystallinity of the systems. The TG analyses of non-isothermal degradation of PHB/ENR-50/MMT nanohybrids showed that the thermal degradation of nanohybrids occurred in two stages. The maximum decomposition temperatures were shifted to lower temperatures upon increasing the MMT content. However, increasing the MMT loading in the PHB/ENR-50 blends gave rise to a catalytic and heat barrier effect. FTIR spectra of the nanohybrids indicate a complicated structure arising from H–bondings and ring opening reactions. The mechanism of formation of the nanohybrid is proposed. 
